# Supplementary material for: Designing strong inducible synthetic promoters in yeasts
Source: Nat Commun. 2024 Dec 19;15:10653. doi: 10.1038/s41467-024-54865-z (PMC11659477; doi:10.1038/s41467-024-54865-z)
Supplement: Supplementary file 1 — Supplementary Information [file 41467_2024_54865_MOESM1_ESM.pdf]

Supplementary information for "**Designing strong inducible synthetic promoters in yeasts**"

- Supplementary Figs. 1–19

Glossary

- *AmpR*, ampicillin resistant gene
- *BsdR,* blastcidin resistant gene
- DAPG, 2,4-diacetylphloroglucinol
- Dox, doxycycline
- EGFP, enhanced green fluorescent protein
- *G418R*, G418 resistant gene
- HSL, *N*-(ketocaproyl)-D,L-homoserine lactone
- *KpARG4*, argininosuccinate lyase gene from *Komagataella phaffii*
- $KpARG4_{962}$, C-terminal 962 bp of *KpARG4* gene
- $KpARG4_{563}$, C-terminal 563 bp of *KpARG4* gene
- *HygR*, Hygromycin resistant gene
- MFα 2×Adv, mating factor alpha (MFα) prepro-leader signal derived from *Saccharomyces cerevisiae* harboring L42S and V50A mutations
- NLS, 7-amino-acid nuclear localization signal from the simian virus 40 large tumor antigen
- *pMB1*, origin of replication for *Escherichia coli*
- RBD^{om}, receptor binding domain of spike protein from a SARS-CoV-2 omicron variant
- $synT_{KpULA}$, terminator region of *KpULA1* gene from *K. phaffii* with a single synthetic AscI site
- $synT_{36177}$, terminator region of *CCA36177* gene from *K. phaffii* with a single synthetic AscI site
- TATA, TATA-box
- T_{38473}, terminator region of *CCA38473* gene from *K. phaffii*
- VP48, three copies of a transcription activating sequence from herpes simplex virus
- *ZeoR*, zeocin resistant gene

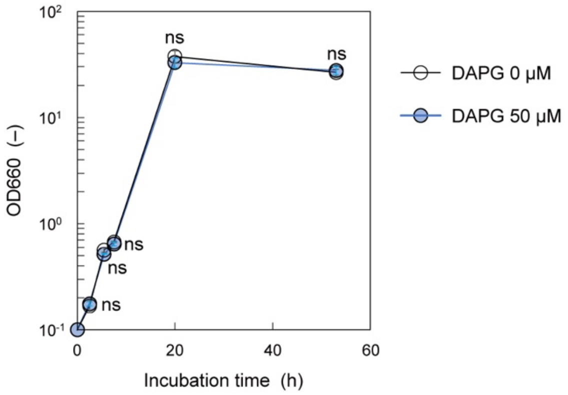

Supplementary Fig. 1. Cell growth of *K. phaffii* strain expressing rPhlTA$_{2\text{-}1E}$ in the presence and absence of 50 µM DAPG. *K. phaffii* strain PpMT146 was grown at 30°C and 170 rpm in 20 mL YPD media with or without 50 µM DAPG (initial OD_{660} = 0.1). OD_{660} was monitored at each time point. Error bars represent the mean ± SD of three independent experiments. ns, not significant. The *p*-values of two-sided paired *t*-test are provided in Source Data.

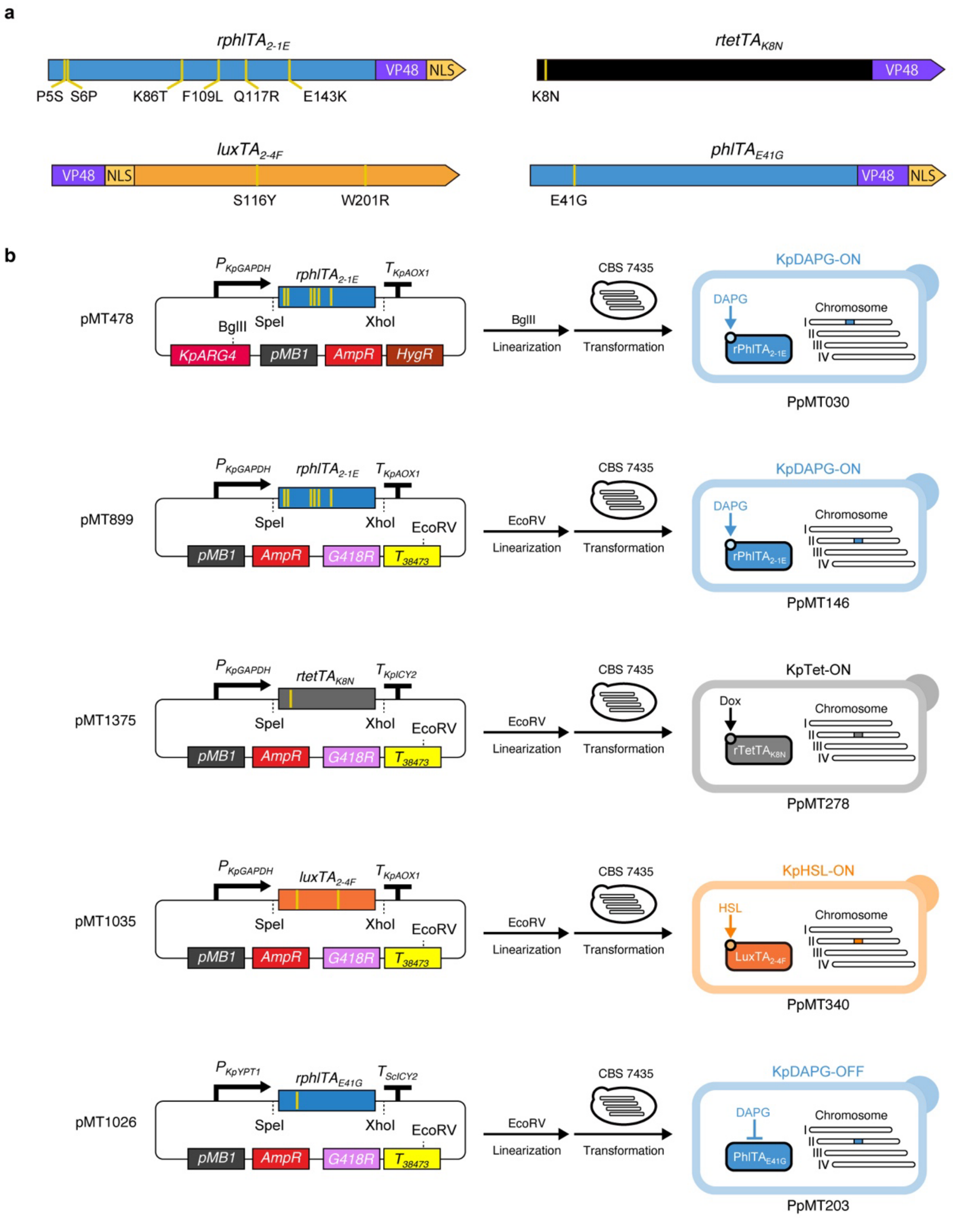

Supplementary Fig. 2. Expression plasmids for the synthetic transcription activators used. a. Illustration of the synthetic transcription activators used in this study. **b.** Schematic for vector maps for plasmids harboring gene encoding synthetic transcription activators and the strain construction using the plasmids. Each plasmid was digested with appropriate restriction enzyme and used for the transformation.

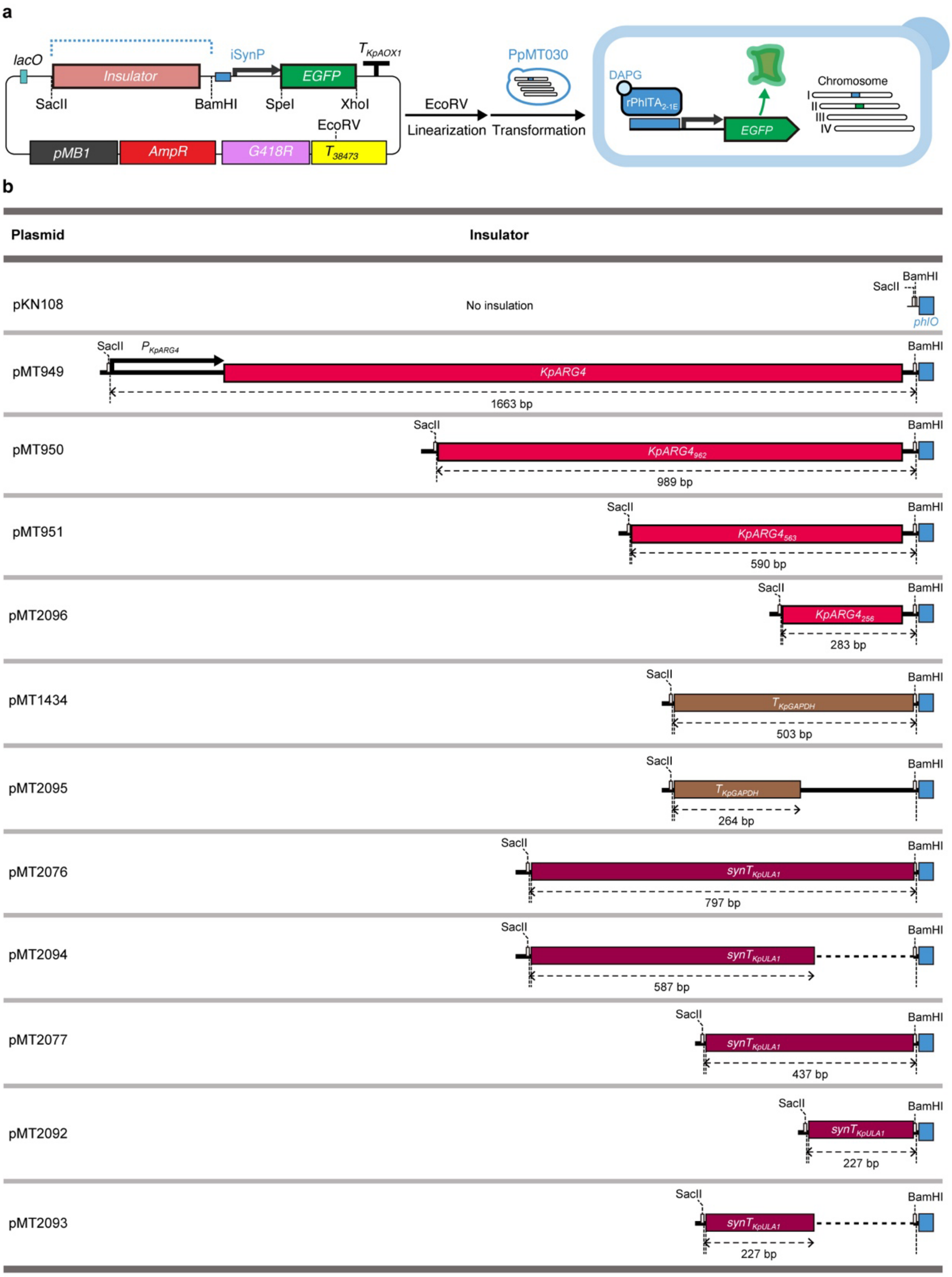

Supplementary Fig. 3. Detailed information for insulation sequences used in Fig. 2. a. Schematic for vector maps for plasmids harboring *KpARG4*, *KpULA1* terminator with AscI insertion (synT_{KpULA1}) or *KpGAPDH* terminator ($T_{KpGAPDH}$) sequences upstream of iSynP and the strain construction using the plasmids. **b.** In-scale schematic of each insulator.

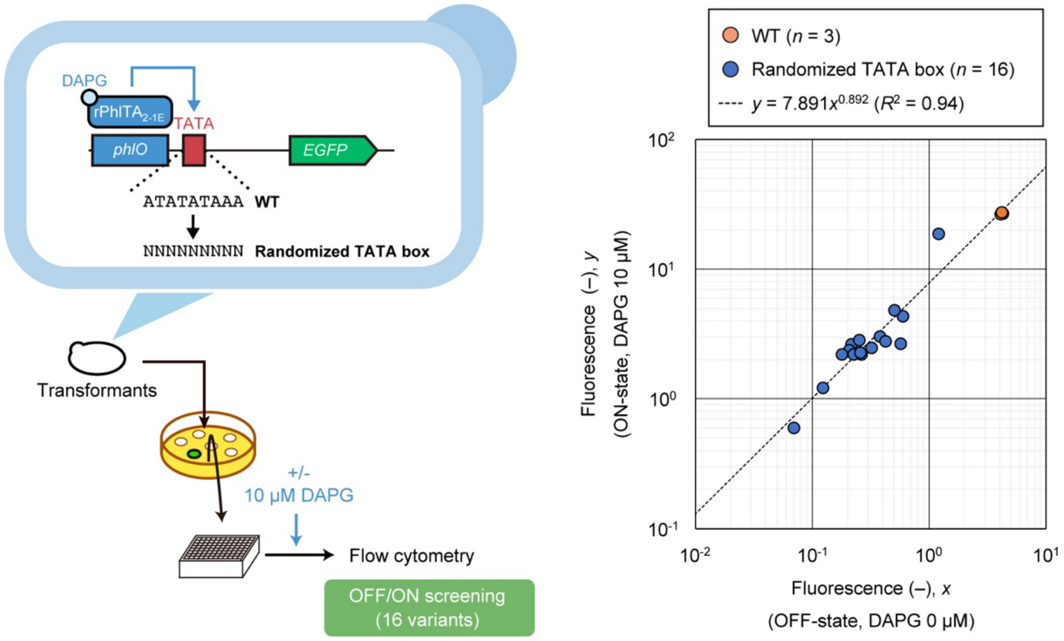

Supplementary Fig. 4. **Leakiness of the prototype DAPG-iSynP with a mutated TATA-box sequence.** The TATA-box sequence within the DAPG-iSynP was randomized by PCR cloning. The resulting mutant plasmid library was linearized and used to transform yeast strain constitutively expressing rPhlTA. Transformants are selected on an agar plate containing G418 and 16 colonies incubated with or without DAPG (10 μM). Cellular EGFP fluorescence was evaluated using flow cytometry as described in the Methods section. ON-state EGFP expression is plotted against OFF-state EGFP expression. Dots represent the individual fluorescence intensity of independent experiments. DAPG, 2,4-diacetylphloroglucinol; EGFP, enhanced green fluorescent protein; eTA, eukaryotic transcriptional activator; TATA, TATA-box.

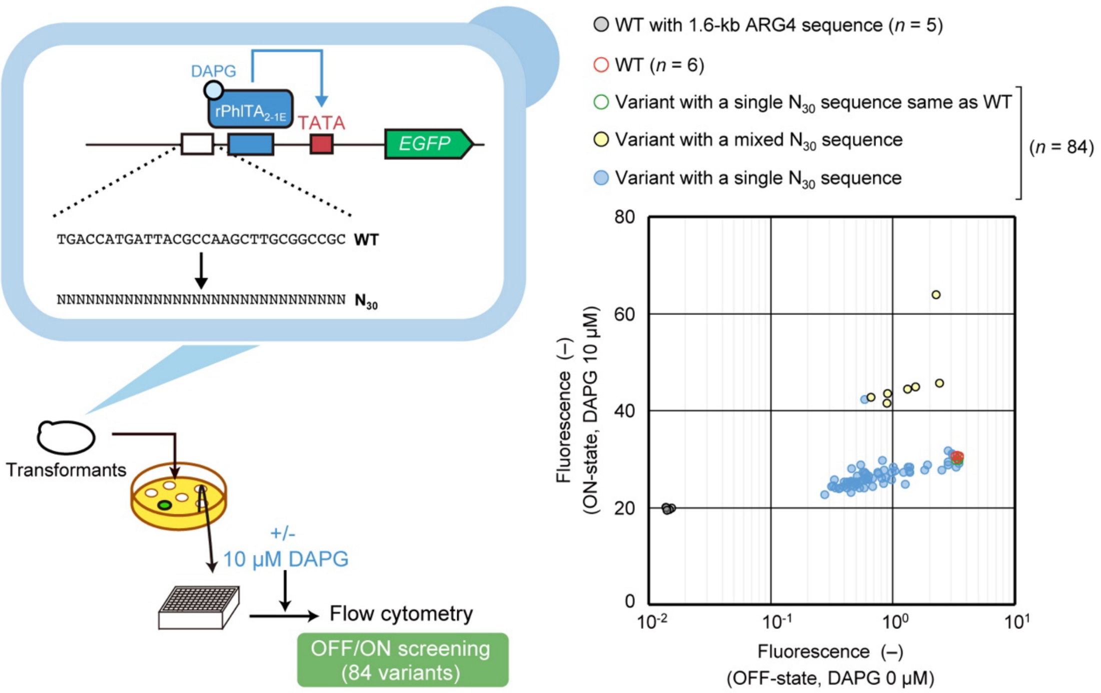

Supplementary Fig. 5. Screening of the upstream 30-bp vector sequence with reduced leakiness from iSynP. A 30-bp sequence upstream of *phlO* was randomized using a randomized primer and cloned into the expression vector. The resulting plasmid was linearized and used to transform the *K. phaffii* strain expressing rPhlTA. Overall, 84 transformants were picked and incubated with or without DAPG (10 µM), followed by flow cytometry analysis to measure cellular EGFP expression as described in the Methods section. ON-state EGFP expression (with 10 µM DAPG) is plotted against OFF-state EGFP expression (without DAPG). We assumed that mutations upstream of the iSynP minimally affect ON-state EGFP expression. Therefore, variants with a >100-fold decrease in ON-state EGFP expression compared to the WT strain were excluded from the analysis. DAPG, 2,4-diacetylphloroglucinol; EGFP, enhanced green fluorescent protein; TATA, TATA-box

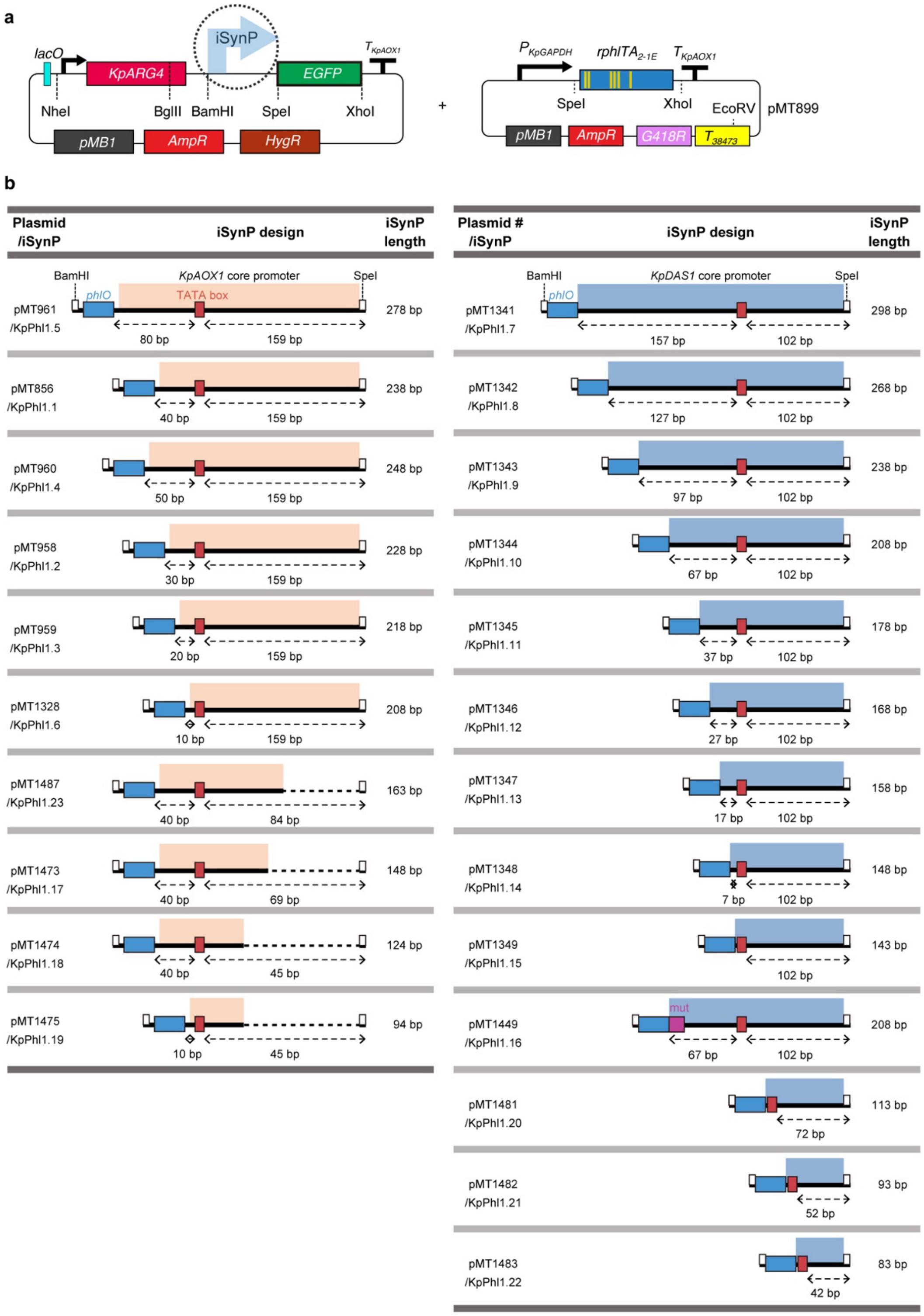

Supplementary Fig. 6. DAPG-inducible synthetic promoters used in Fig. 3. a. Schematic for plasmids harboring different iSynP and the strain construction using the plasmids. **b.** In-scale schematic for iSynPs; KpPhl1.16, a 15-bp sequence downstream of *phlO* was replaced with sequence from −173 to −187 region of *KpDAS1* promoter; this replacement is indicated as purple box.

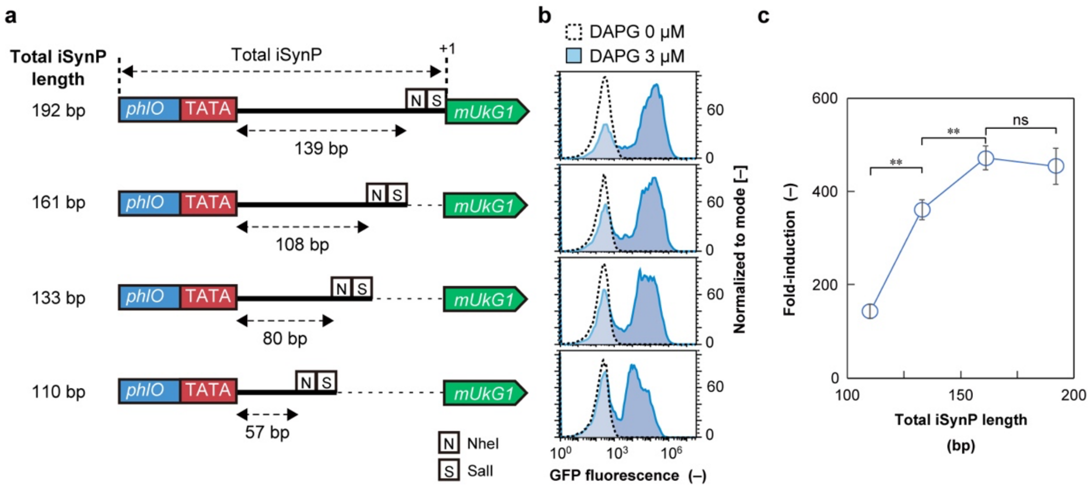

Supplementary Fig. 7. Construction of minimal iSynPs in *S. cerevisiae*. a. Schematic illustration of DAPG-iSynPs for *S. cerevisiae* with different spacer lengths between the TATA-box and the start codon. **b**. DAPG-induced GFP expression of the *S. cerevisiae* strains harboring the DAPG-iSynPs were measured Flow-cytometry. **c.** Fold-induction was calculated by dividing GFP fluorescence in the presence of 3 μM DAPG by the uninduced GFP fluorescence in its absence, as measured by flow cytometry. mUkG1, monomeric variant of umikinoko-green 1; N, NheI; S, SalI. TATA, TATA-box. Error bars represent the mean ± SD of four independent experiments. The double asterisk represents p <0.01. ns, not significant. The p-values of two-sided Welch's t-test are provided in Source Data. ns, not significant.

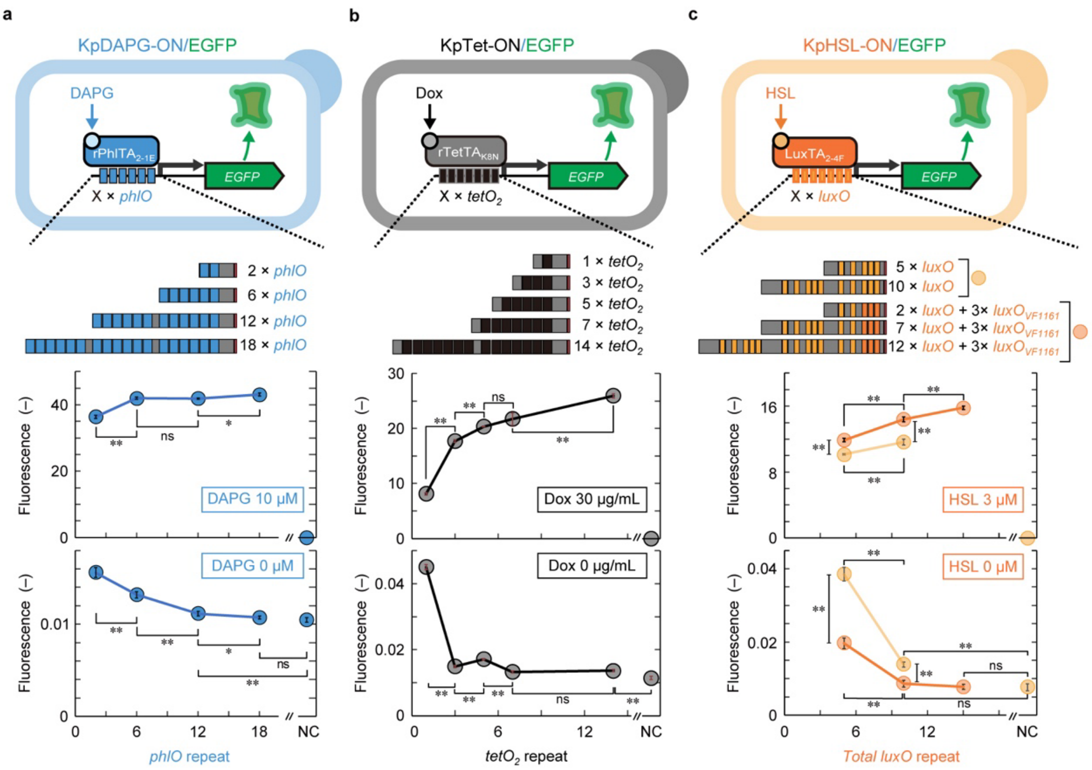

Supplementary Fig. 8. Impact of repeat number of bacterial operators on induced and uninduced EGFP expression of different synthetic inducible switches (Fig. 4). The promoter architecture of these iSynPs is shown in Supplementary Figs. 8–10. Error bars represent the mean ± SD of four (a and b) or six (c) independent experiments. The single and double asterisk represents p <0.05 and p <0.01, respectively. The p-values of two-sided Welch's t-test are provided in Source Data. TATA-box and spacer sequence are indicated in red and gray boxes, respectively. DAPG, 2,4-diacetylphloroglucinol; Dox, doxycycline; HSL, *N*-(ketocaproyl)-D,L-homoserine lactone. NC, negative control (without reporter plasmid); ns, not significant.

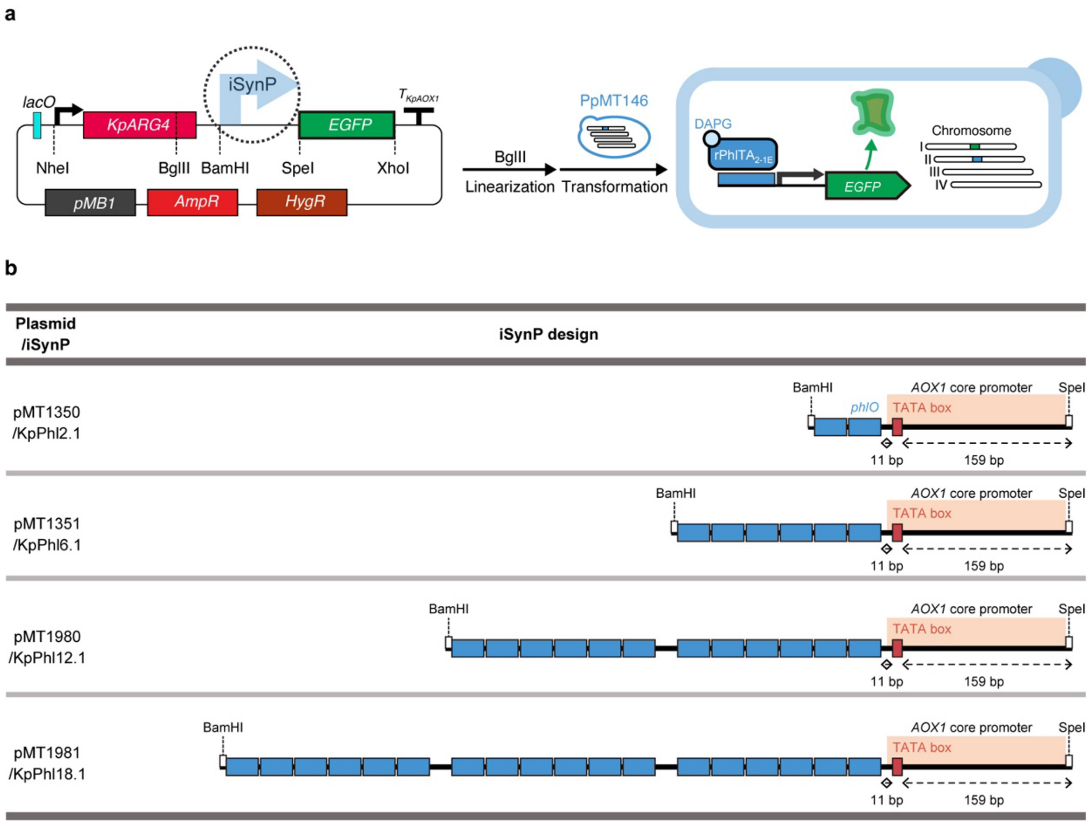

Supplementary Fig. 9. DAPG-inducible synthetic promoters used in Fig. 4a. a. Schematic for plasmids harboring different iSynP and the strain construction using the plasmids. **b.** In-scale schematic for iSynPs.

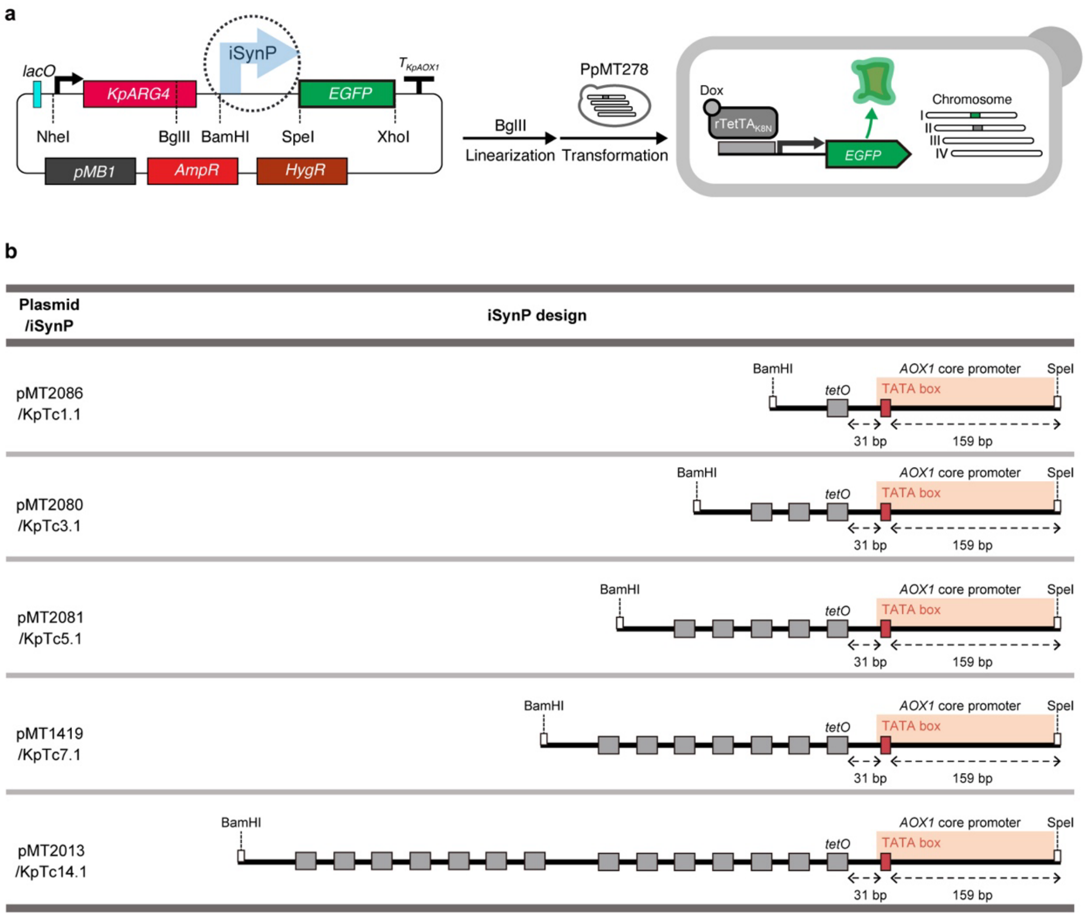

Supplementary Fig. 10. Dox-inducible synthetic promoters used in Fig. 4b. a. Schematic for plasmids harboring different iSynP and the strain construction using the plasmids. **b.** In-scale schematic for iSynPs.

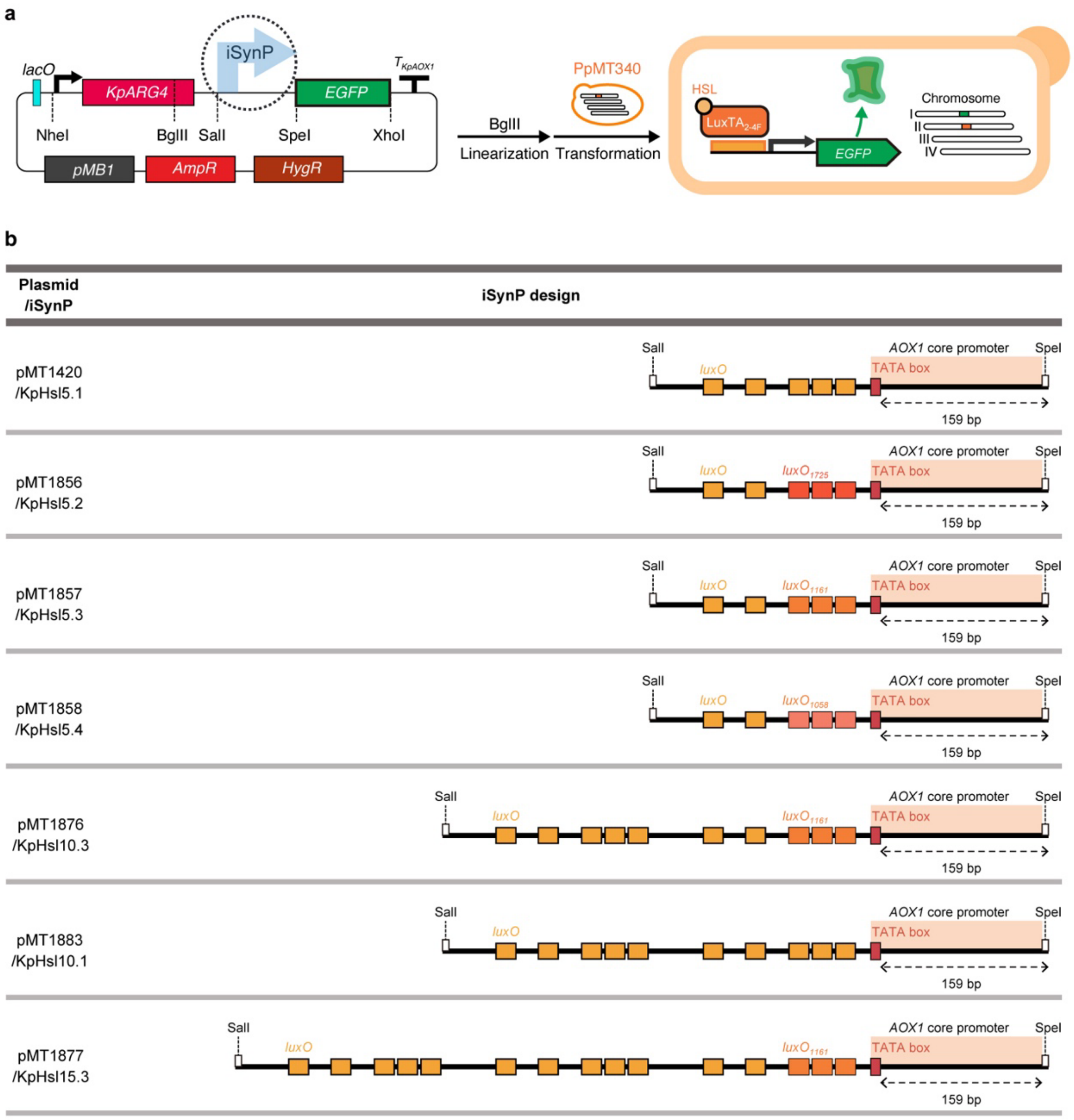

Supplementary Fig. 11. HSL-inducible synthetic promoters used in Fig. 4c. a. Schematic for plasmids harboring different iSynP and the strain construction using the plasmids. **b.** In-scale schematic for iSynPs.

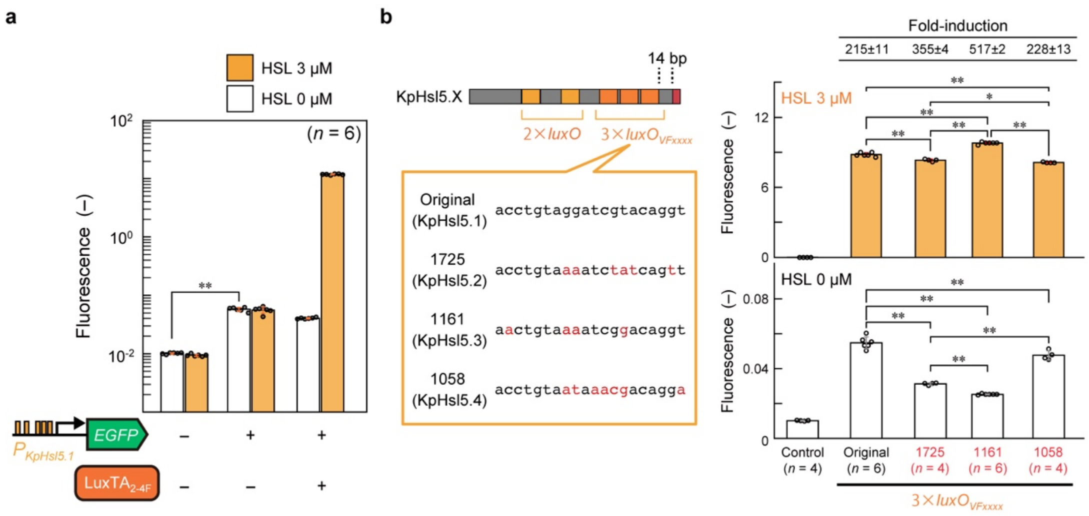

Supplementary Fig. 12. Optimizing HSL-iSynP. a. The cryptic activation of the *luxO* operator sequence was detected when fused directly to the TATA-box even without LuxTA expression. **b**. HSL-dependent gene expression using different *luxO* sequences from *Aliivibrio fischeri* (formerly *Vibrio fischeri*). Individual fluorescence intensity from independent experiments is shown with dots, and the average intensity is represented by the bar graph. Error bars represent the mean ± SD based on multiple independent experiments. Sample sizes are shown in the figures. The single and double asterisk represents $p < 0.05$ and $p < 0.01$, respectively. The *p*-values of two-sided Welch's *t*-test are provided in Source Data. When comparing on- and off-state fluorescence, a paired *t*-test was used.EGFP, enhanced green fluorescent protein; HSL, *N*-(ketocaproyl)-D,L-homoserine lactone.

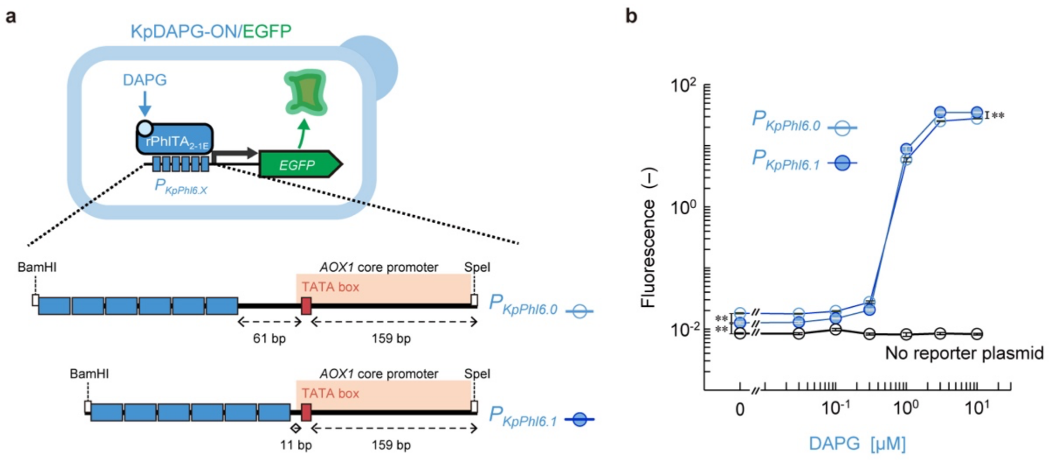

Supplementary Fig. 13. Switching performance of DAPG-ON system using iSynPs with different spacer length. a. Schematic illustration of the strain with KpDAPG-ON system, in which rPhlTA$_{2\text{-}1E}$ binds to $P_{KpPhl6.0}$ and $P_{KpPhl6.1}$ with 61- and 11-bp spacer sequence between *phlO* repeats and TATA-box to activate EGFP expression. **b**. Dose-response curves for the two KpDAPG-ON systems ($n = 4$). The double asterisk represents p <0.01. The p-values of two-sided Welch's t-test are provided in Source Data.

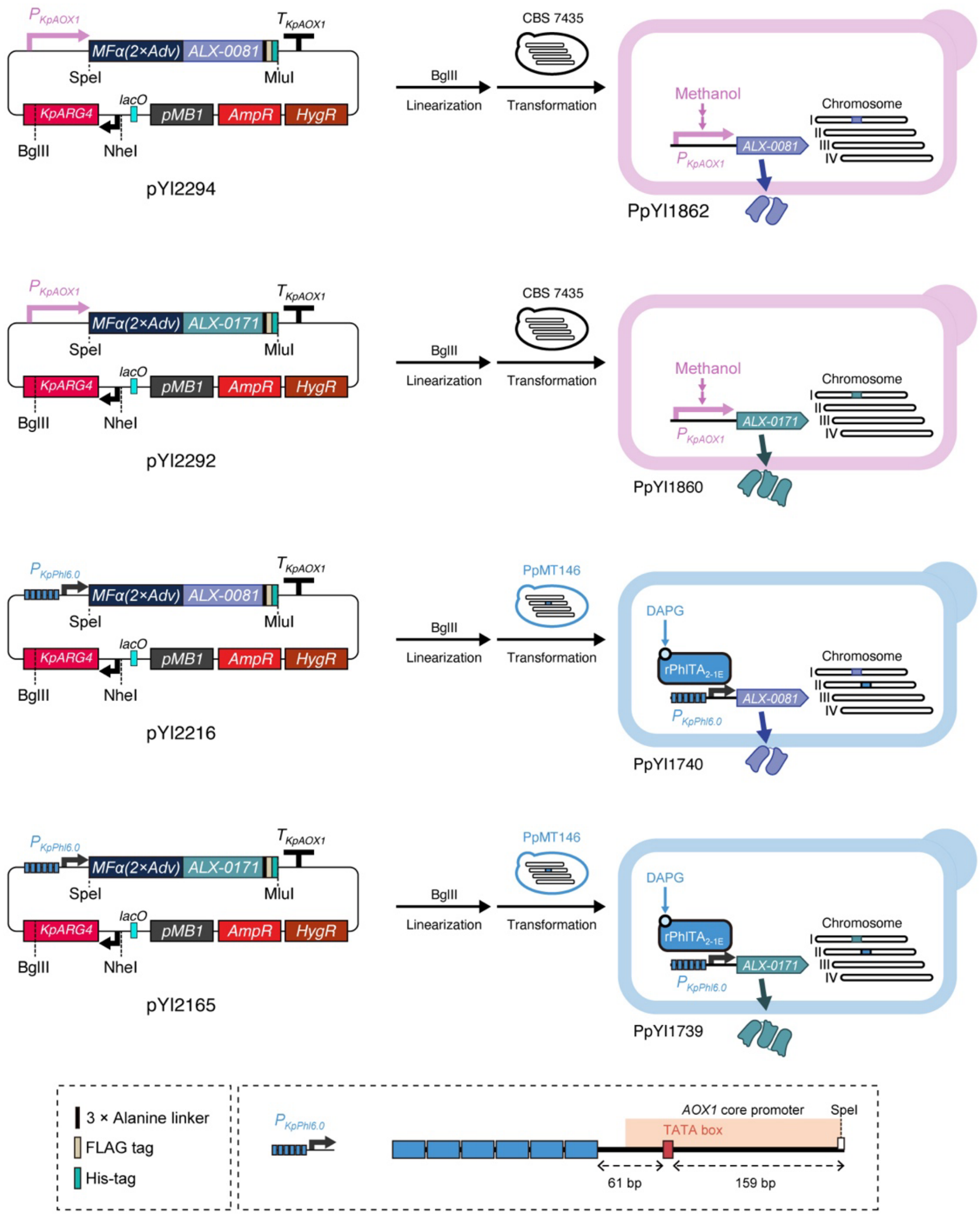

Supplementary Fig. 14. Construction of *K. phaffii* strain harboring nanobody-encoding plasmids used for inducible nanobody production in Fig. 5a. Schematic for vector maps for plasmids harboring gene encoding nanobodies and the strain construction using the plasmids. Each plasmid was digested with appropriate restriction enzyme and used for the transformation.

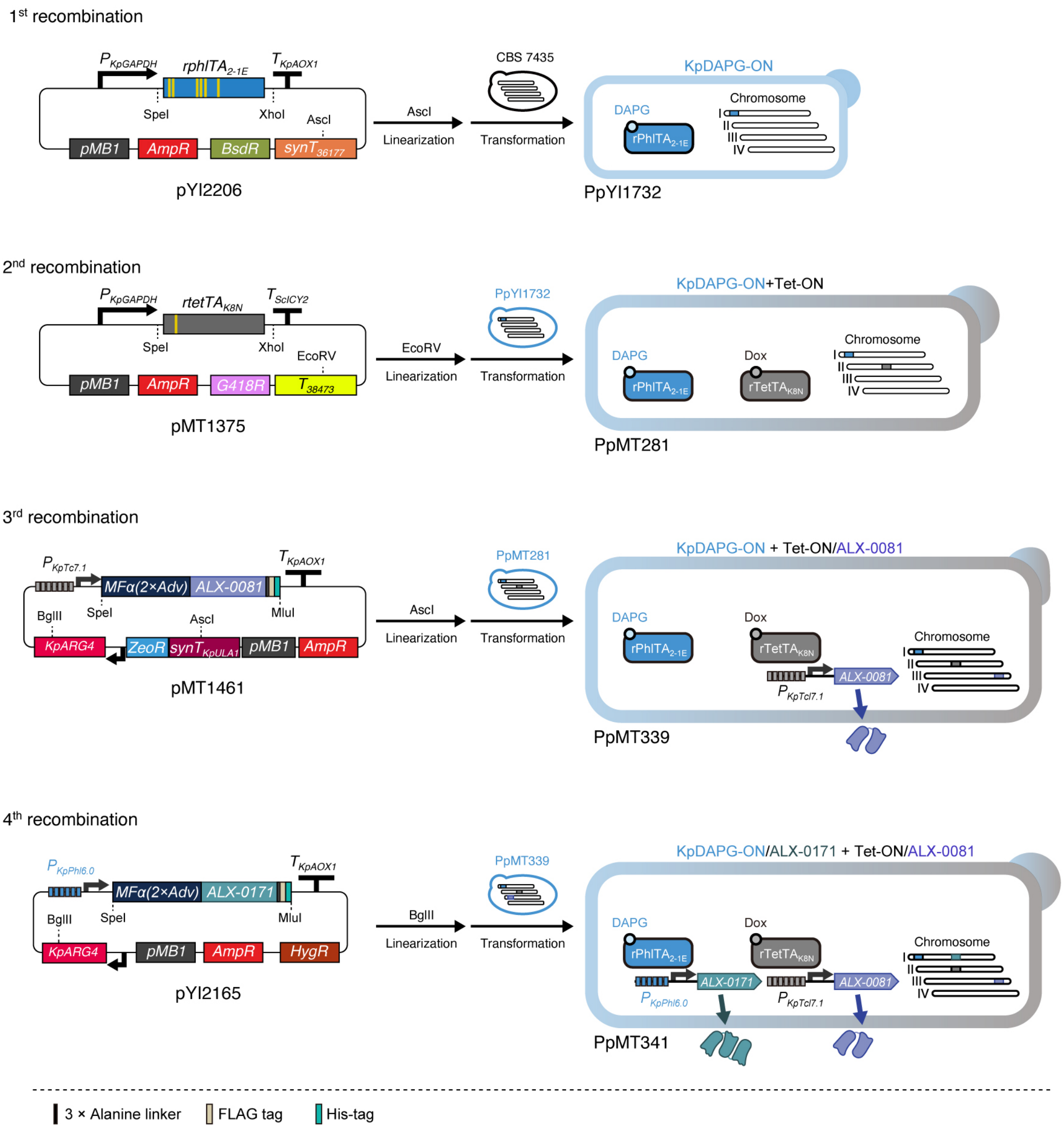

Supplementary Fig. 15. Four-step construction of *K. phaffii* strain harboring nanobody-encoding plasmids used for selective nanobody production in Fig. 5b. Schematic for vector maps for plasmids harboring gene encoding synthetic transcription activators and nanobodies, and the strain construction using the plasmids. Each plasmid was digested with appropriate restriction enzyme and used for the transformation.

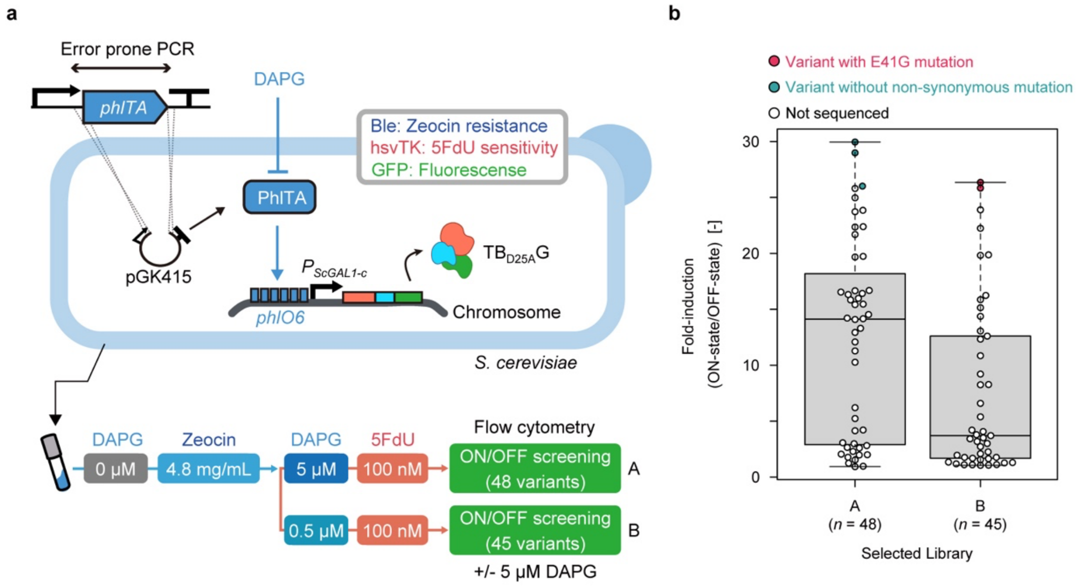

Supplementary Fig. 16. Directed evolution of DAPG-deactivatable sTA in *S. cerevisiae*. a. Schematic of the directed evolution cycle. Details are described in the Methods section. **b**. Fold induction of the selected PhlTA variant. Data for sequenced PhlTA variants are indicated as colored dots. $TB_{D25A}G$, fusion protein of herpes simplex virus thymidine kinase, Zeocin-resistance protein D25A mutant, and a green fluorescent protein (monomeric umikinoko green 1).

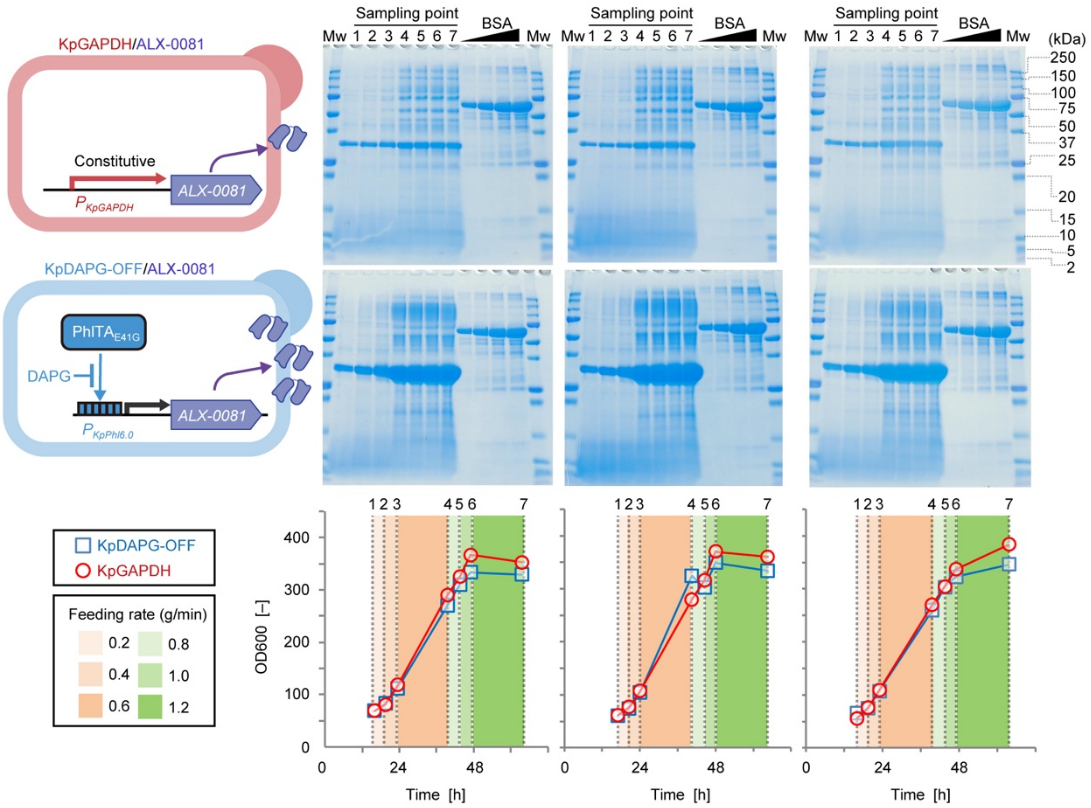

Supplementary Fig. 17. SDS-PAGE analysis and fermentation conditions for the experiment shown in Fig. 6b. Feeding solution was added to the media at a rate shown in the legend. BSA used as the standard in the concentrations 125, 250, 500, and 750 mg/L from left to right. BSA, Bovine serum albumin; Mw, molecular weight marker.

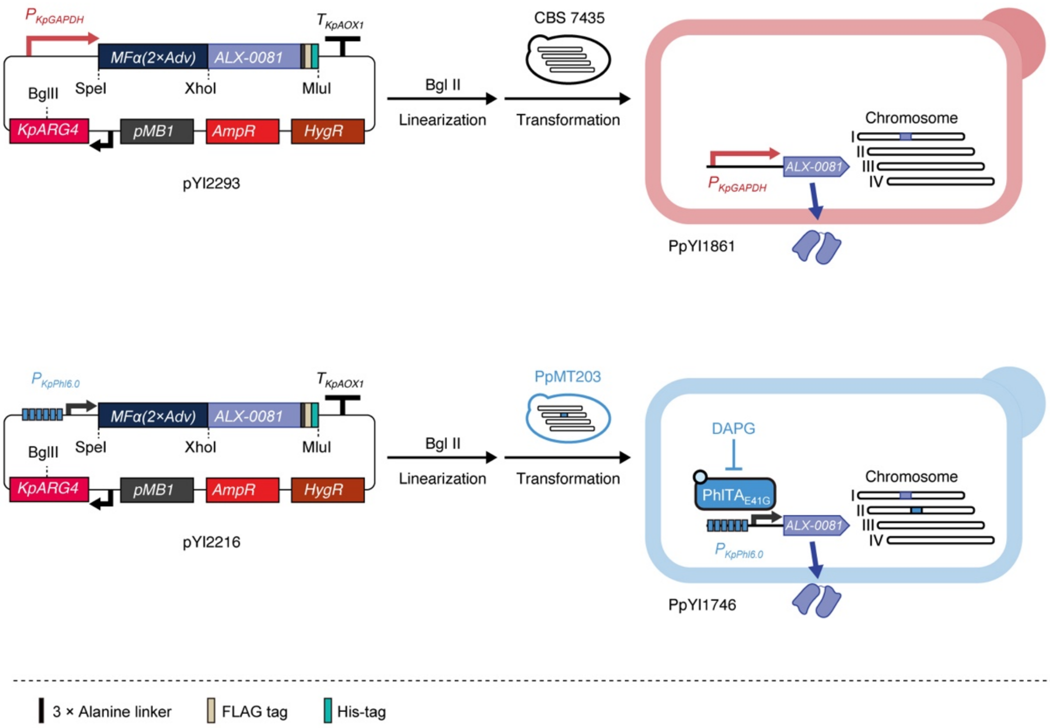

Supplementary Fig. 18. Construction of *K. phaffii* strain harboring nanobody-encoding plasmids used for large-scale nanobody secretive production in Fig. 6b. Schematic for vector maps for plasmids harboring gene encoding nanobodies and the strain construction using the plasmids. Each plasmid was digested with appropriate restriction enzyme and used for the transformation.

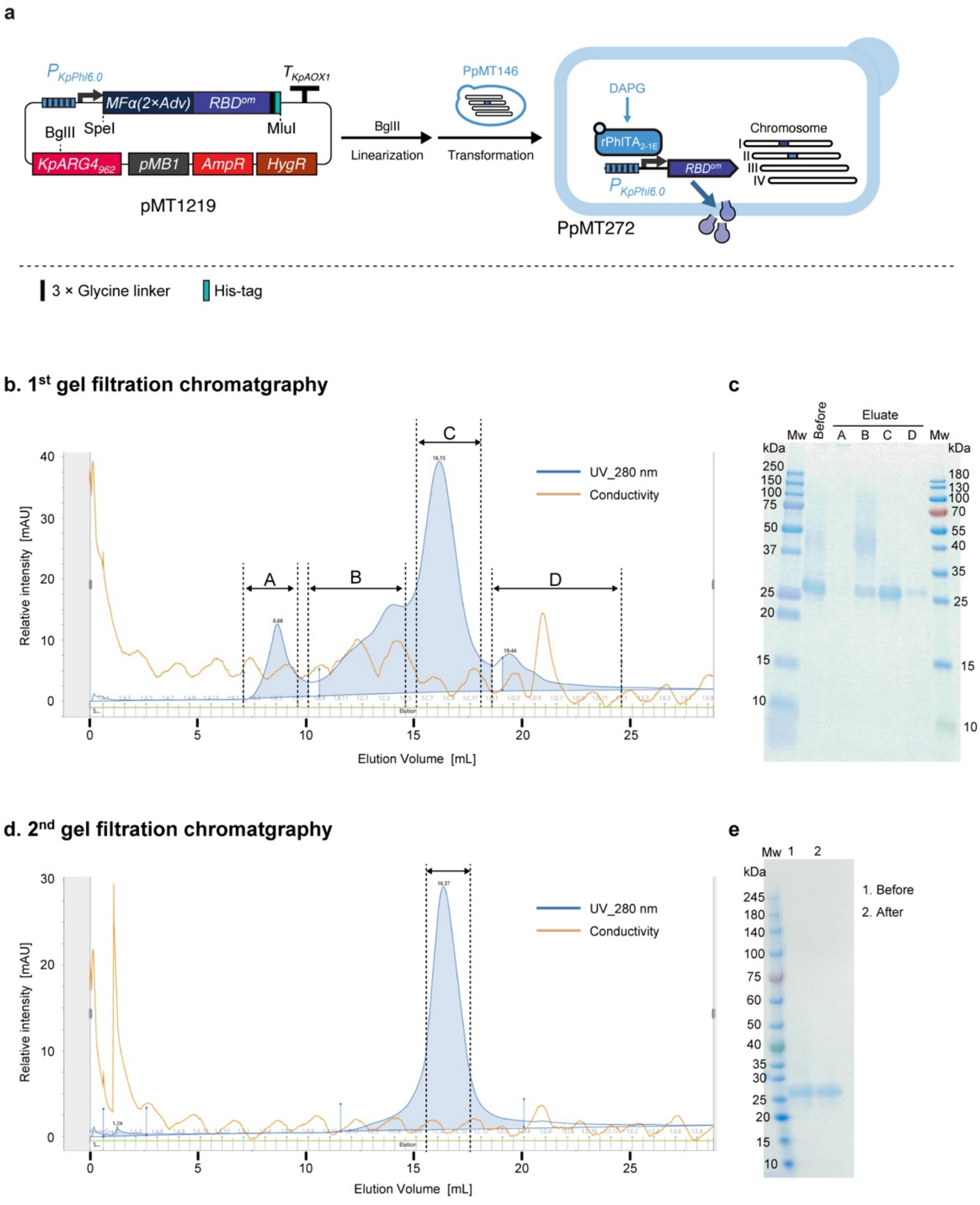

Supplementary Fig. 19. Purification of RBD^{om} from yeast supernatant using gel filtration chromatography. a. Construction of *K. phaffii* strain harboring RBD^{om}-encoding plasmids used for DAPG-inducible RBD^{om} secretive production. **b-e**. His-tag purified RBD^{om} was subjected to gel filtration chromatography and subsequently concentrated twice. **b**,**d**. Chromatogram of RBD^{om} purified by 1st (**b**) and 2nd (**d**) gel filtration chromatography using Superdex 200 increase. The recombinant protein before and after each gel filtration step was analyzed by SDS-PAGE (**c**,**e**). Fractions indicated by dashed lines and double arrow were collected for further use. Mw, molecular weight marker.
