# Supplementary material for: Designing strong inducible synthetic promoters in yeasts
Source: Nat Commun. 2024 Dec 19;15:10653. doi: 10.1038/s41467-024-54865-z (PMC11659477; doi:10.1038/s41467-024-54865-z)
Supplement: Supplementary file 2 — Description of Additional Supplementary Files [file 41467_2024_54865_MOESM2_ESM.pdf]

Description of Additional Supplementary Files

File Name: Supplementary Data 1

Description: Plasmids used in this study

File Name: Supplementary Data 2

Description: *Komagataella phaffii* strains used in this study

File Name: Supplementary Data 3

Description: *Saccharomyces cerevisiae* strains used this study

File Name: Supplementary Data 4

Description: Primers used in this study

File Name: Supplementary Data 5

Description: DNA parts used in this study

File Name: Supplementary Data 6

Description: Inducible synthetic promoters used in this study

File Name: Supplementary Data 7

Description: Insulator sequences used in this study
